# Supplementary material for: Cyanocobalamin prevents cardiomyopathy in type 1 diabetes by modulating oxidative stress and DNMT-SOCS1/3-IGF-1 signaling
Source: Commun Biol. 2021 Jun 23;4:775. doi: 10.1038/s42003-021-02291-y (PMC8222371; doi:10.1038/s42003-021-02291-y)
Supplement: Supplementary file 2 — Supplemental Information [file 42003_2021_2291_MOESM2_ESM.pdf]

Supplementary Information

Supplemental Table 1. Primers and probes for quantification of mRNA with real-time quantitative reverse transcription-PCR.

Gene Symbol	Primer/Probe	Sequence
Actb	(Fwd primer)	5'-AAGAGCTATGAGCTGCCTGA-3'
	(Rev primer)	5'-ACGGATGTCAACGTCACACT-3'
	(Probe)	5'-FAM-CACTATTGGCAACGAGCGGTTCCG-Tamra-3'
Socs3	(Fwd primer)	5'-CTTTGTAGACTTCACGGCTG-3'
	(Rev primer)	5'-TATGGTCCCCGAGGCGAG-3'
	(Probe)	5'-FAM-AGTGGCTCGCGCTGCGCCCAGA-Tamra-3'
Myh6	(Fwd primer)	5'-CCAAGTTCGACAAGATCGAG-3'
	(Rev primer)	5'-CCGAGTAGGTATAGATCATC-3'
	(Probe)	5'-FAM-TGGCCATGCTGACCTTCCTGCA-Tamra-3'
Myh7	(Fwd primer)	5'-TTGAGAATCCAAGGCTCAGC-3'
	(Rev primer)	5'-TGAGGTCAAAGGGCCTGGT-3'
	(Probe)	5'-FAM-TGCCTCCAGCCTCTCCTTCTCAGA-Tamra-3'
Trp53	(Fwd primer)	5'-TGCTCCGATGGTGATGGCCT-3'
	(Rev primer)	5'-TGTGGCGAAAAGTCTGCCTG-3'
	(Probe)	5'-FAM-TTGGCACCAATGTCCCGGCT-Tamra-3'

The primers and probes for *Igf1, Socs1, Dnmt1, Dnmt3a* and *Dnmt3b* were from TaqMan™ Gene Expression Assays (Thermo Fisher Scientific).

Suppl. Fig. 1: Oral high dose B12 improves cardiac function in *Elmo1$^{H/H}$ Ins2$^{Akita/+}$* mice.

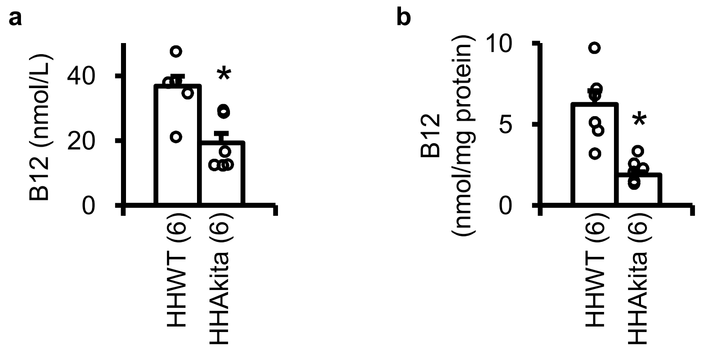

a Plasma levels of B12 in HHWT and HHAkita mice. **b** Cardiac levels of B12 in HHWT and HHAkita mice. Numbers in the axis label indicate the number of animals studied. HHWT, *Elmo1$^{H/H}$ Ins2$^{+/+}$*; HHAkita, *Elmo1$^{H/H}$ Ins2$^{Akita/+}$*; *, *P* < 0.05 vs. HHWT by Student;s t-test; TL, tibia length.

Suppl. Fig. 2: Effects of orally administered B12 (10 mg/kg/d) on the cardiac function in the *Elmo1$^{+/+}$ Ins2$^{Akita/+}$* mice at age 16 weeks.

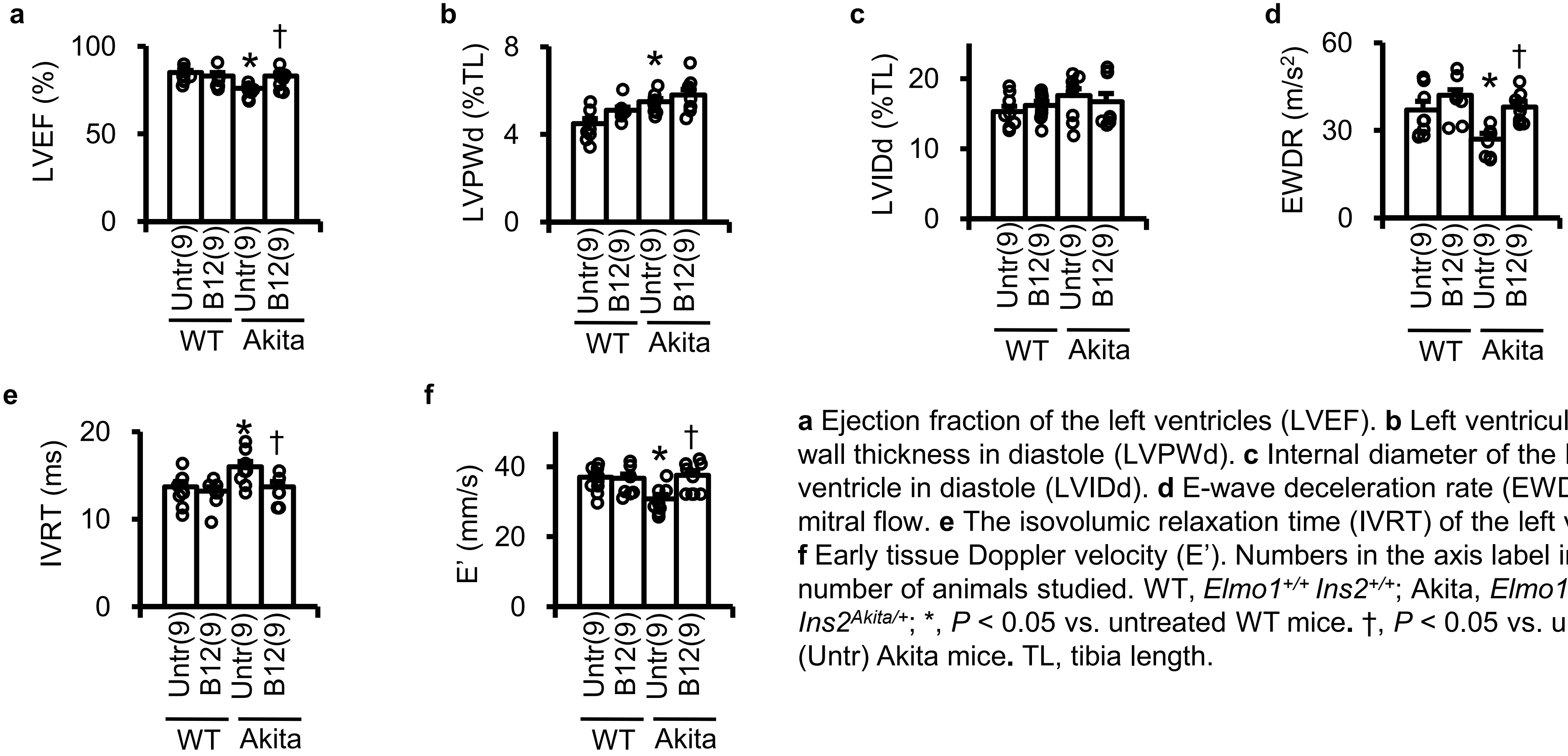

a Ejection fraction of the left ventricles (LVEF). **b** Left ventricular posterior wall thickness in diastole (LVPWd). **c** Internal diameter of the left ventricle in diastole (LVIDd). **d** E-wave deceleration rate (EWDR) of the mitral flow. **e** The isovolumic relaxation time (IVRT) of the left ventricle. **f** Early tissue Doppler velocity (E'). Numbers in the axis label indicate the number of animals studied. WT, *Elmo1$^{+/+}$ Ins2$^{+/+}$*; Akita, *Elmo1$^{+/+}$ Ins2$^{Akita/+}$*; *, *P* < 0.05 vs. untreated WT mice**.** †, *P* < 0.05 vs. untreated (Untr) Akita mice**.** TL, tibia length.

Suppl. Fig. 3: Cardioprotective effects of B12 (10 mg/kg/d) were significantly greater than the other antioxidants N-acetyl-L-cysteine (NAC; 1000 mg/kg/d) and tempol (Tem; 200 mg/kg/d) in *Elmo1*$^{H/H}$*Ins2*$^{Akita/+}$ mice.

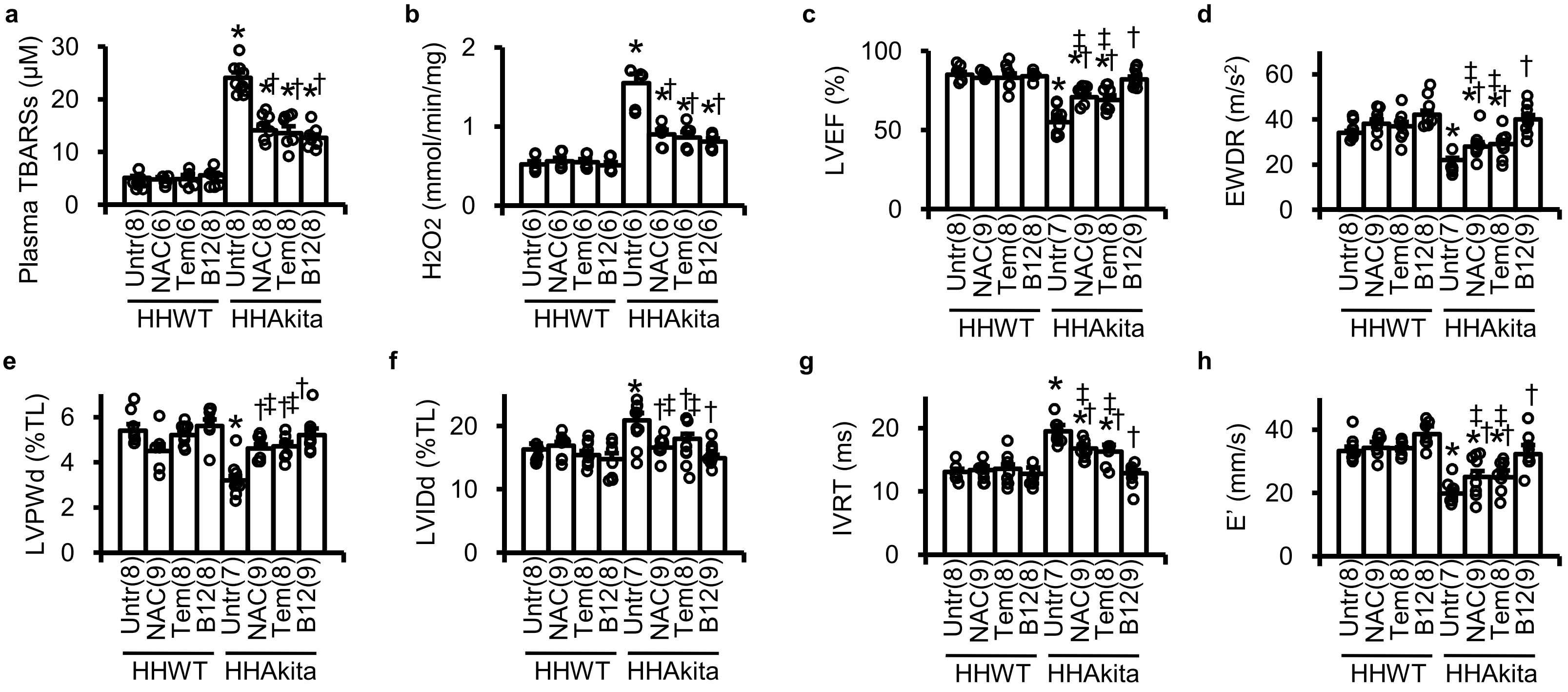

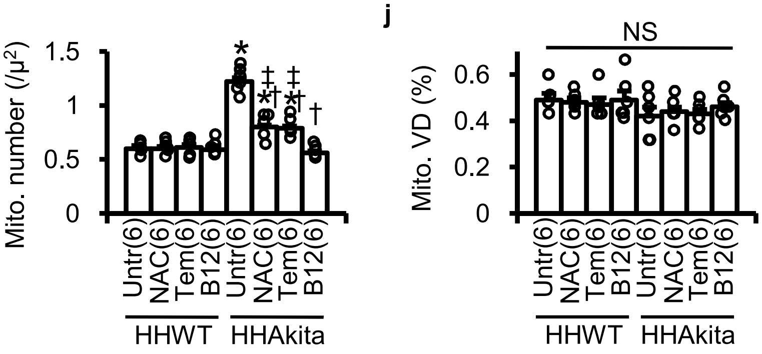

a Plasma levels of thiobarbiturate-reactive substances (TBARSs). **b** cardiac H2O2 release. **c** Ejection fraction of the left ventricles (LVEF). **d** E-wave deceleration rate (EWDR) of the mitral flow. **e** Left ventricular posterior wall thickness in diastole (LVPWd) as % of TL. **f** Internal diameter of the left ventricle in diastole (LVIDd) as % of TL. **g** Isovolumic relaxation time (IVRT) of the left ventricle. **h** Early tissue Doppler velocity (E'). **i** Number of mitochondria (Mito.). **j** Volume density (VD) of mitochondria (Mito). Numbers in the axis label indicate the number of animals studied. HHWT, *Elmo1*$^{H/H}$ *Ins2*$^{+/+}$; HHAkita, *Elmo1*$^{H/H}$ *Ins2*$^{Akita/+}$; *, $P< 0.05$ vs. untreated (Untr) HHWT mice by Tukey-Kramer Honestly Significant Differences test after one-way ANOVA; †, $P < 0.05$ vs. untreated HHAkita mice; ‡, $P < 0.05$ vs. B12-treated HHAkita mice; TL, tibia length.

Suppl. Fig. 4: **Expression of apoptosis- and cardiomyopathy- associated genes and the parameters related to insulin-like growth factor (IGF)-1 signaling.**

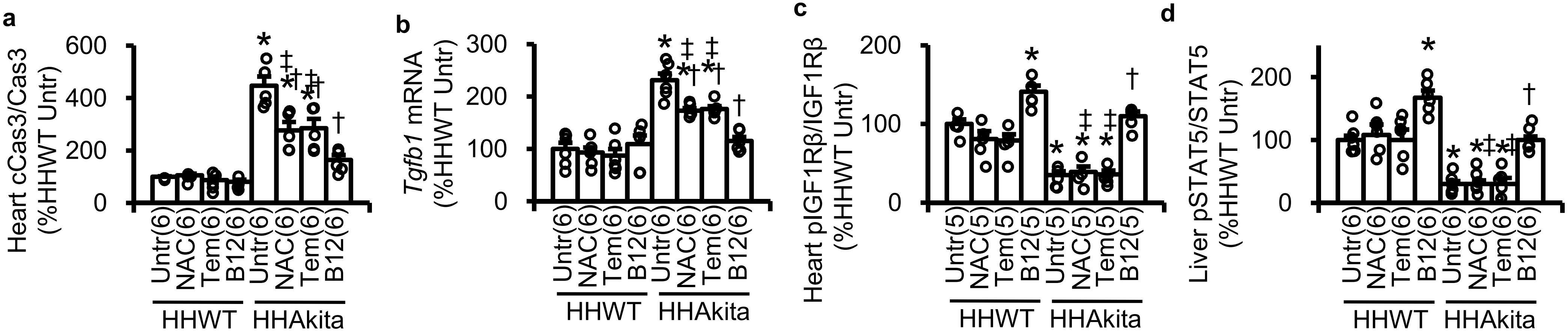

a Cardiac cleaved caspase 3 (cCas3) levels normalized by total Cas3 levels. **b** Cardiac *Tgfb1* mRNA levels normalized by β-actin mRNA levels. **c** Cardiac levels of phosphorylated IGF-1 receptor β (pIGF1Rβ) normalized by total IGF1Rβ levels. **d** Hepatic phosphorylated pSTAT5 levels normalized by total STAT5 levels. The number of animals analyzed is given in each figure. HHWT, *Elmo1*$^{H/H}$ *Ins2*$^{+/+}$; HHAkita, *Elmo1*$^{H/H}$ *Ins2*$^{Akita/+}$; *, $P < 0.05$ vs. untreated (Untr) HHWT mice by Tukey-Kramer Honestly Significant Differences test after one-way ANOVA; †, $P < 0.05$ vs. untreated HHAkita mice; ‡, $P < 0.05$ vs. B12-treated HHAkita mice; NS, not significant.

Suppl. Fig. 5: DNMT's-SOCS1/3-IGF1 signaling mediates the cardioprotective effects of B12 in *Elmo1$^{H/H}$Ins2$^{Akita/+}$* (HHAkita) mice.

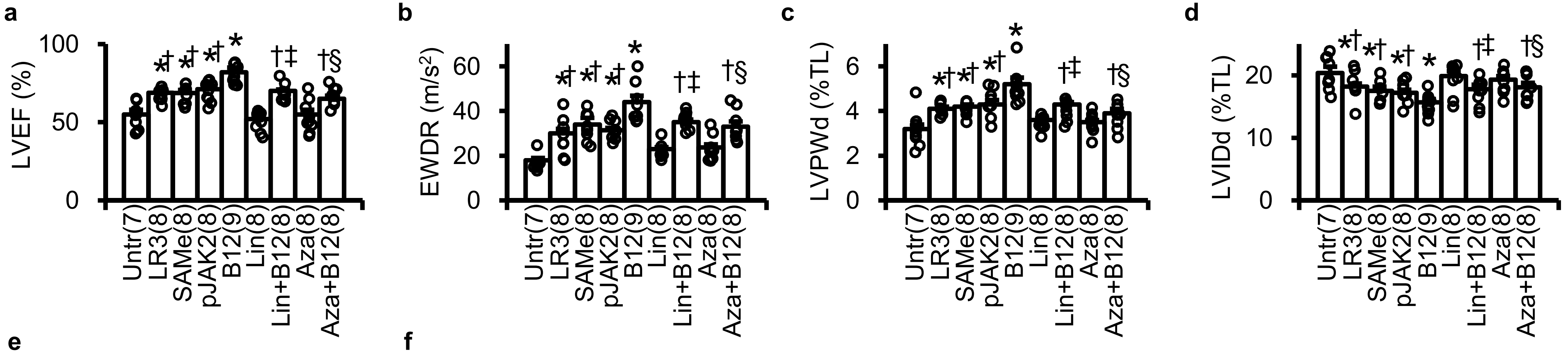

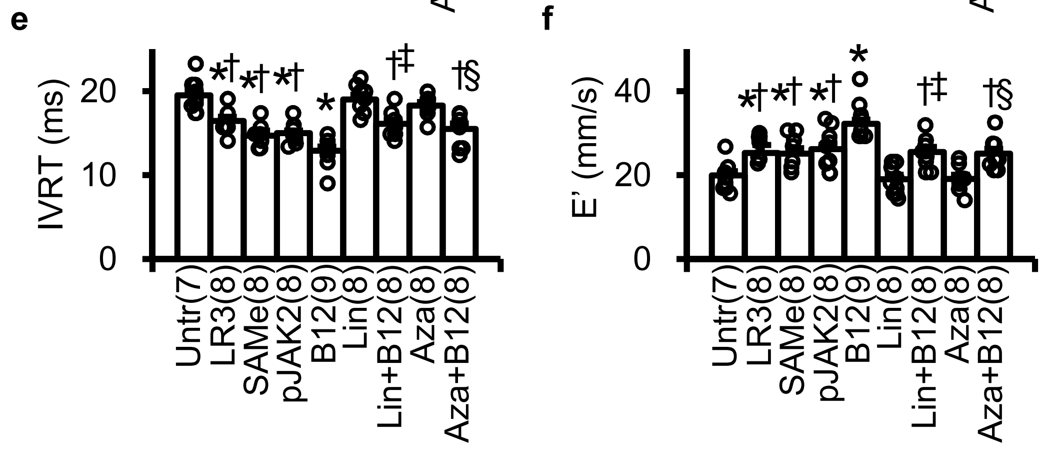

a-f Effects of a long-acting IGF-1 analog IGF-1 Long R3 (LR3; 0.3 mg/kg/d IP), S-adenosylmethionine (SAMe; 200 mg/kg/d PO), a SOCS1/3 inhibitor pJAK2(1001-1013) (pJAK2; 10 mg/kg/d IP), B12 alone, and a DNMT inhibitor 5-aza-2'-deoxycytidine (Aza; 20 mg/kg/d IP) and an IGF-1 receptor inhibitor linsitinib (Lin; 50 mg/kg/d PO) with and without B12 on **a** LVEF, **b** EWDR, **c** LVPWd, **d** LVIDd, **e** IVRT and **f** E'. The number of animals analyzed is given in each figure.*, $P < 0.05$ vs. Untr; †, $P < 0.05$ vs. B12; ‡, $P < 0.05$ vs. Lin; §, $P < 0.05$ vs. Aza; by Tukey-Kramer Honestly Significant Differences test. TL, tibia length.

Suppl. Fig. 6: DNMT's-SOCS1/3-IGF1 signaling mediates the cardioprotective effects of B12 in *Elmo1$^{H/H}$ Ins2$^{Akita/+}$* (HHAkita) mice.

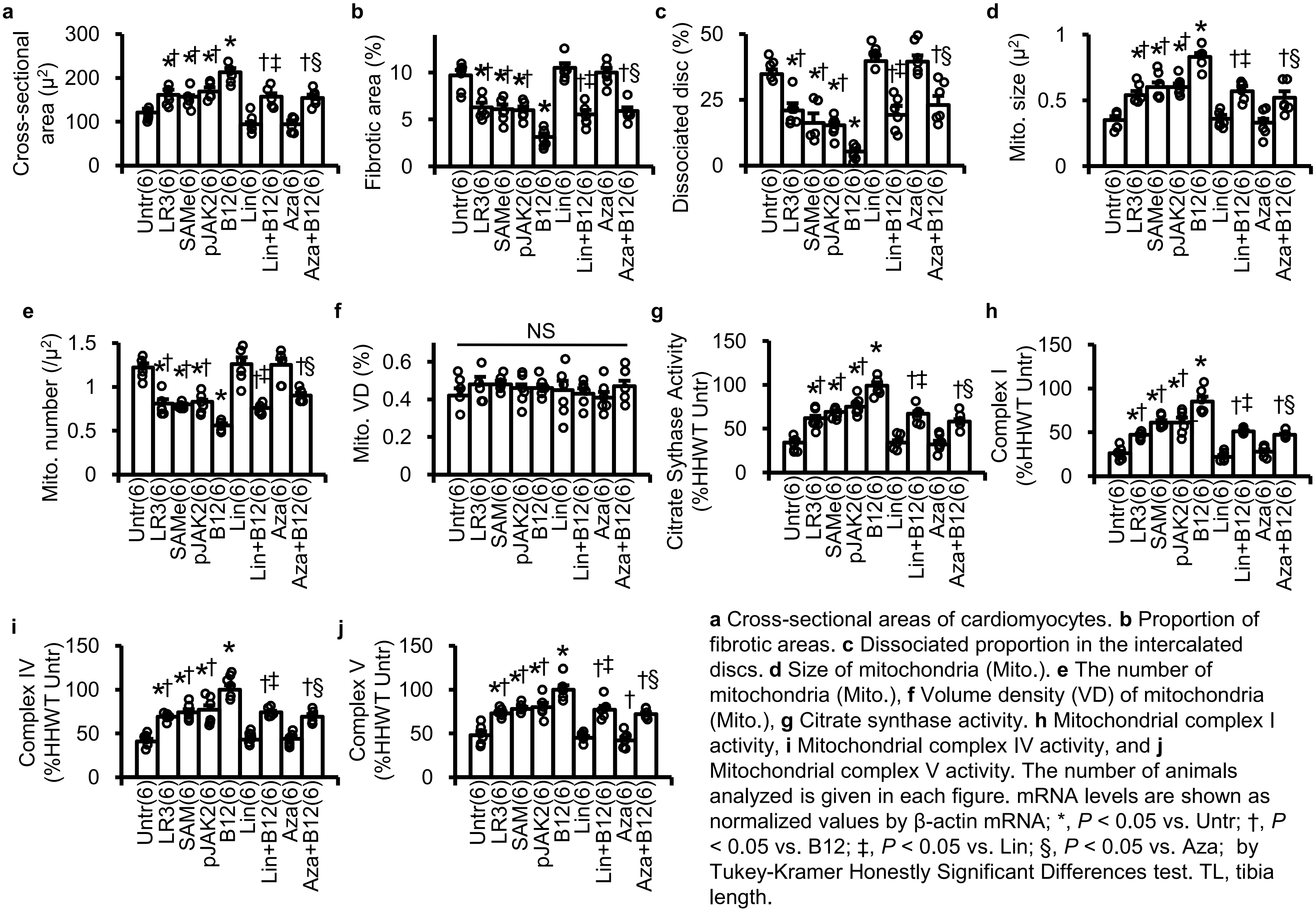

a Cross-sectional areas of cardiomyocytes. **b** Proportion of fibrotic areas. **c** Dissociated proportion in the intercalated discs. **d** Size of mitochondria (Mito.). **e** The number of mitochondria (Mito.), **f** Volume density (VD) of mitochondria (Mito.), **g** Citrate synthase activity. **h** Mitochondrial complex I activity, **i** Mitochondrial complex IV activity, and **j** Mitochondrial complex V activity. The number of animals analyzed is given in each figure. mRNA levels are shown as normalized values by β-actin mRNA; *, P < 0.05 vs. Untr; †, P < 0.05 vs. B12; ‡, P < 0.05 vs. Lin; §, P < 0.05 vs. Aza; by Tukey-Kramer Honestly Significant Differences test. TL, tibia length.

Suppl. Fig. 7: Uncropped immunoblot images.

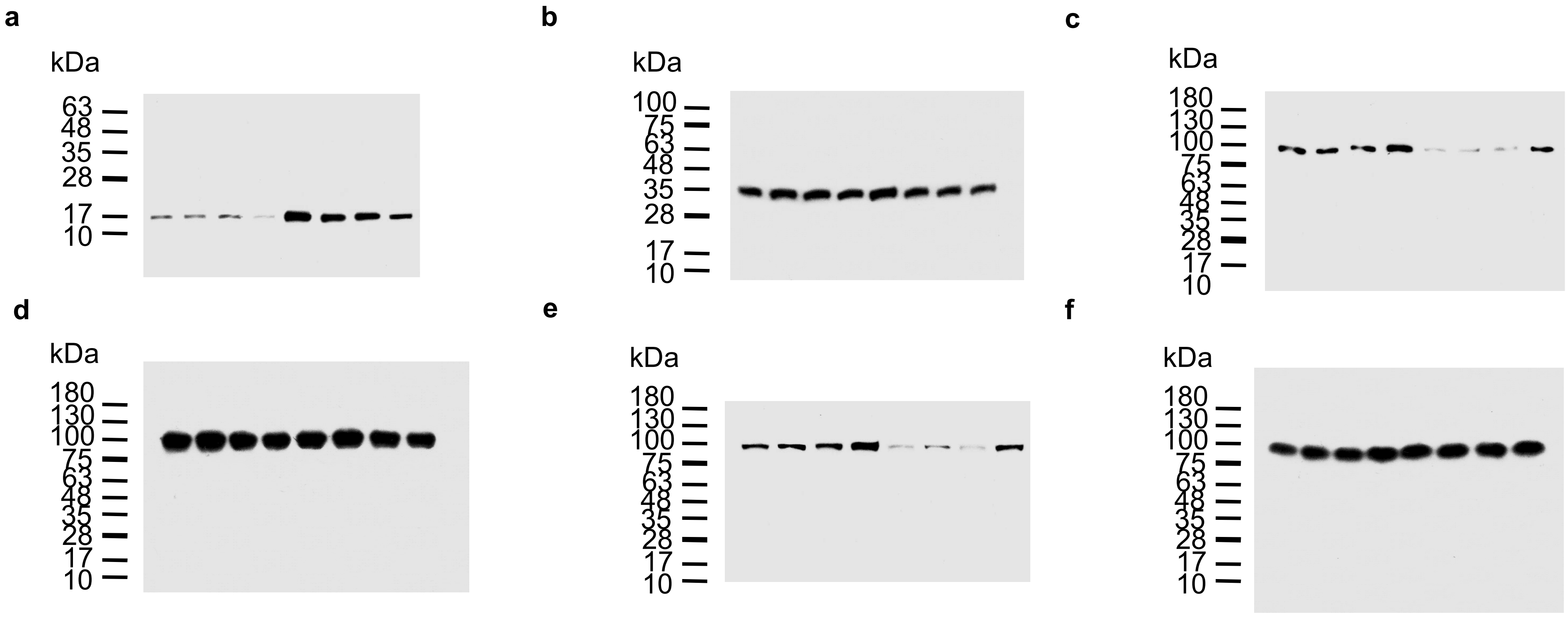

a Cardiac cleaved caspase 3 (cCas3). **b** Cardiac total caspase 3 (Cas3). **c** Cardiac phosphorylated IGF-1 receptor β (pIGF1Rβ). **d** Cardiac total IGF1Rβ. **e** Hepatic phosphorylated pSTAT5. **f** Hepatic total STAT5.
