# Supplementary material for: Cyanocobalamin prevents cardiomyopathy in type 1 diabetes by modulating oxidative stress and DNMT-SOCS1/3-IGF-1 signaling
Source: Commun Biol. 2021 Jun 23;4:775. doi: 10.1038/s42003-021-02291-y (PMC8222371; doi:10.1038/s42003-021-02291-y)
Supplement: Supplementary file 3 — Description of Supplementary Files [file 42003_2021_2291_MOESM3_ESM.pdf]

Description of Additional Supplementary Files

File name: Supplementary data 1

Description: Basic characteristics in experimental groups.

File name: Supplementary data 2

Description: Source Data.
