# Supplementary material for: Cyanocobalamin prevents cardiomyopathy in type 1 diabetes by modulating oxidative stress and DNMT-SOCS1/3-IGF-1 signaling
Source: Commun Biol. 2021 Jun 23;4:775. doi: 10.1038/s42003-021-02291-y (PMC8222371; doi:10.1038/s42003-021-02291-y)
Supplement: Supplementary file 6 — Reporting Summary [file 42003_2021_2291_MOESM6_ESM.pdf]

nature research

Corresponding author(s): Masao Kakoki

Last updated by author(s): May 19, 2021

Reporting Summary

Nature Research wishes to improve the reproducibility of the work that we publish. This form provides structure for consistency and transparency in reporting. For further information on Nature Research policies, see our Editorial Policies and the Editorial Policy Checklist.

Statistics

For all statistical analyses, confirm that the following items are present in the figure legend, table legend, main text, or Methods section.

n/a	Confirmed	
☐	☒	The exact sample size (*n*) for each experimental group/condition, given as a discrete number and unit of measurement
☐	☒	A statement on whether measurements were taken from distinct samples or whether the same sample was measured repeatedly
☐	☒	The statistical test(s) used AND whether they are one- or two-sided<br>*Only common tests should be described solely by name; describe more complex techniques in the Methods section.*
☐	☒	A description of all covariates tested
☐	☒	A description of any assumptions or corrections, such as tests of normality and adjustment for multiple comparisons
☐	☒	A full description of the statistical parameters including central tendency (e.g. means) or other basic estimates (e.g. regression coefficient) AND variation (e.g. standard deviation) or associated estimates of uncertainty (e.g. confidence intervals)
☐	☒	For null hypothesis testing, the test statistic (e.g. *F*, *t*, *r*) with confidence intervals, effect sizes, degrees of freedom and *P* value noted<br>*Give P values as exact values whenever suitable.*
☒	☐	For Bayesian analysis, information on the choice of priors and Markov chain Monte Carlo settings
☒	☐	For hierarchical and complex designs, identification of the appropriate level for tests and full reporting of outcomes
☒	☐	Estimates of effect sizes (e.g. Cohen's *d*, Pearson's *r*), indicating how they were calculated

Our web collection on statistics for biologists contains articles on many of the points above.

Software and code

Policy information about availability of computer code

Data collection	Vevo 2100 (VisualSonics)
Data analysis	JMP14 (SAS Institute Inc.), ImageJ (NIH)

For manuscripts utilizing custom algorithms or software that are central to the research but not yet described in published literature, software must be made available to editors and reviewers. We strongly encourage code deposition in a community repository (e.g. GitHub). See the Nature Research guidelines for submitting code & software for further information.

Data

Policy information about availability of data

All manuscripts must include a data availability statement. This statement should provide the following information, where applicable:

- Accession codes, unique identifiers, or web links for publicly available datasets
- A list of figures that have associated raw data
- A description of any restrictions on data availability

Provide your data availability statement here.

Field-specific reporting

Life sciences study design

All studies must disclose on these points even when the disclosure is negative.

Sample size	Because a priori power analyses indicated the sample size between 6 and 10 is needed in most experiments, this range of number of mice was used.
Data exclusions	No data were excluded.
Replication	Reproducibility including biologically independent sample sizes is stated in each figure legend.
Randomization	Mice were randomly allocated into experimental groups.
Blinding	The investigators were blinded when they read histological and functional parameters.

Reporting for specific materials, systems and methods

We require information from authors about some types of materials, experimental systems and methods used in many studies. Here, indicate whether each material, system or method listed is relevant to your study. If you are not sure if a list item applies to your research, read the appropriate section before selecting a response.

Materials & experimental systems

n/a	Involved in the study	
☐	☒	Antibodies
☒	☐	Eukaryotic cell lines
☒	☐	Palaeontology and archaeology
☐	☒	Animals and other organisms
☒	☐	Human research participants
☒	☐	Clinical data
☒	☐	Dual use research of concern

Methods

n/a	Involved in the study	
☒	☐	ChIP-seq
☒	☐	Flow cytometry
☒	☐	MRI-based neuroimaging

Antibodies

Antibodies used	Y1135-phosphorylated IGF-1 receptor β (pIGF1Rβ; Rabbit mAb; DA7A8; Cell Signaling Technology), IGF-1 receptor β (IGF1Rβ; Rabbit pAb; 3027; Cell Signaling Technology), Y694-phosphorylated signal transducer and activator of transcription 5 (pSTAT5; Rabbit mAb; C11C5; Cell Signaling Technology), total STAT5 (Rabbit mAb; D206Y; Cell Signaling Technology), cleaved caspase 3 (Rabbit mAb; 9664; Cell Signaling Technology), total caspase 3 (Rabbit mAb; 8610; Cell Signaling Technology), and β-actin (HRP-conjugated Rabbit mAb; 3683; Cell Signaling Technology).
Validation	Generally, the antibodies from Cell Signaling Technology is highly specific and give few non-specific bands. The pictures of typical blots are presented in their websites, and the usage of these antibodies was cited in a number of published articles (at least 44, 244, 170, 32, 2599, 856, 189 articles, respectively).

Animals and other organisms

Policy information about studies involving animals; ARRIVE guidelines recommended for reporting animal research

Laboratory animals	Elmo1(H/H) Ins2(Akita/+) and Elmo1(H/H) Ins2(+/+) male mice at age of 24 weeks.
Wild animals	None.
Field-collected samples	None.
Ethics oversight	All mice were kept under husbandry conditions conforming to the National Institutes of Health Guideline for Use and Care of Experimental Animals as approved by the Institutional Animal Care and Use Committee.

Note that full information on the approval of the study protocol must also be provided in the manuscript.
